# A Comparison of the Effects of Benzalkonium Chloride on Ocular Surfaces between C57BL/6 and BALB/c Mice

**DOI:** 10.3390/ijms18030509

**Published:** 2017-02-26

**Authors:** Qian Yang, Yafang Zhang, Xiuping Liu, Nan Wang, Zhenyu Song, Kaili Wu

**Affiliations:** 1 Zhongshan Ophthalmic Center, State Key Laboratory of Ophthalmology, Sun Yat-sen University, Guangzhou 510060, China; muyangmay@126.com (Q.Y.); LXP__2171@126.com (X.L.); yankewangnan@126.com (N.W.); songzhenyu08@sina.com (Z.S.); 2 Department of Ophthalmology, Hubei University of Science and Technology, Xianning 437100, China; zhangyafang709@163.com

**Keywords:** benzalkonium chloride, cornea, conjunctiva, mouse, C57BL/6, BALB/c

## Abstract

Models of benzalkonium chloride (BAC)-induced ocular disruption have been created and are widely used in various animals. This study aimed to compare the effects of BAC on the ocular surfaces of C57BL/6 and BALB/c mice. C57BL/6 and BALB/c mice were treated separately with BAC eye-drops at different concentrations. Eyes were evaluated by scoring epithelial disruption, corneal opacity and neovascularization in vivo, and by histological assays with hematoxylin/eosin (H/E) and periodic acid-Schiff stainings and by determining the expression of inflammatory factors in vitro on Days 7 and 14. The in vivo corneal epithelial disruption, corneal edema/opacity and neovascularization, which were in accordance with the results of the H/E staining and peaked at Day 7, were observed in a dose-dependent manner in the BAC-treated mice, with more severe signs in the C57BL/6 mice than the BALB/c mice. The loss of conjunctival goblet cells in the conjunctivas and the increasing expression of monocyte chemoattractant protein 1 (MCP-1), growth-regulated protein alpha (GROa) and macrophage inflammatory protein-1 alpha (MIP-1a) in the corneas were found in a dose-dependent manner in both strains of mice. Topical application of BAC can dramatically disrupt the ocular surfaces of C57BL/6 and BALB/c mice, and the disruptions were much more severe in the C57BL/6 mice that received high doses of BAC.

## 1. Introduction

Benzalkonium chloride (BAC), a quaternary ammonium cationic surface-acting agent that dissolves bacterial walls and membranes, is commonly used as a preservative in ophthalmic preparations [1]. Over recent years, experimental and clinical studies have revealed that the long-term use of topical BAC can induce ocular surface changes that are dose- and time-dependent [2,3,4,5]. For example, a clinical survey revealed a high prevalence of dry eye in glaucoma patients who used drops with preservatives, and the prevalence increased with the number of eye drops applied (e.g., approximately a 40% prevalence of dry eye in patients who used two or more drugs vs. 11% in patients receiving one type of eye drop) [1,6]. Removal of BAC from timolol can improve the function of the corneal epithelial barrier and reduce patient complaints [7]. Additionally, significant increases in the conjunctival expression of inflammatory factors, e.g., C-X3-C motif chemokine 1 (CX3CL1), interleukins, C-C chemokine receptor 4 (CCR4) and C-C chemokine receptor 5 (CCR5), have been observed in patients treated with BAC-preserved eye drops over long periods of time [8,9]. These clinical studies have revealed an increasing incidence of adverse events with BAC and have demonstrated that the withdrawal of preservatives reduces these effects. 

The in vivo toxicity of BAC results in inflammation and congestion, tear film instability (i.e., a decrease of break time for tears), loss of conjunctival goblet cells, corneal epithelial desquamation, erosions, ulceration and corneal neovascularization, as well as many other signs in mice [3,10], rats [11], cats [12], rabbits [13,14], guinea pigs [15], and dogs [16]. However, few experimental studies have explored the mechanisms of BAC-induced damage to the ocular surface in addition to its detergent properties. The experiments involving cultured conjunctival and corneal cells demonstrated that BAC-induced changes in cell viability/apoptosis were associated with the production of inflammatory cytokines, DNA damage, intracellular reactive oxygen species and signaling pathways [2,17,18]. In vivo animal experiments have revealed that BAC induces high expression of pro-inflammatory mediators and infiltration of inflammatory cells in the cornea and conjunctiva [1,10,19].

BAC accumulation induces a reduction in mucins and an alteration of the lipid layer, leading to impairments of the tear film with tear instability and excessive evaporation, which are hallmarks of dry eye disease [20]. Due to increasing awareness of the toxicity of BAC to ocular surfaces, BAC has recently been used by various research groups worldwide to induce dry eye models in different animals [4,10,14,21]. Of these dry eye models, two strains of mice, BALB/c and C57BL/6, have been frequently used in studies from different groups in which the BAC eye drops were topically applied in a similar manner. For example, Liu and colleagues in Xiamen, China and Yang and coworkers in Gangneung, Korea developed a dry eye model using BALB/c mice [10,22,23,24]. Barabino and colleagues in Genoa, Italy and Park and coworkers in Daegu, Korea used C57BL/6 mice [3,25]. Some other researchers have used Kunming mice [26] and transgenic mice (CX3CR1 gfp/gfp on C57BL/6) [9]. Typically, these dry eye mouse models were induced by administering 0.2% BAC eye drops two to four times per day and observing the mice for 7–14 days. However, higher (0.3%) or lower (0.02%, four weeks of follow up) concentrations of BAC were used in some studies [26,27]. These different strains of mice may differ in their resistance to BAC damage. However, the above studies successfully induced dry eye using nearly the same treatments and obtained similar results, although they did not pay close attention to the differences between the animal species. 

Thus, we designed this study to determine if there were differences in the ocular surface alterations following regular BAC administration between BALB/c and C57BL/6 mice, two mouse strains that are frequently used in the study of BAC-induced dry eye. We aimed to validate this model of dry eye and determine which strain of mouse is more suitable for dry eye research and other studies.

## 2. Results

### 2.1. In Vivo Ocular Surface Manifestation in Two Strains of Mice

**Figure 1:**
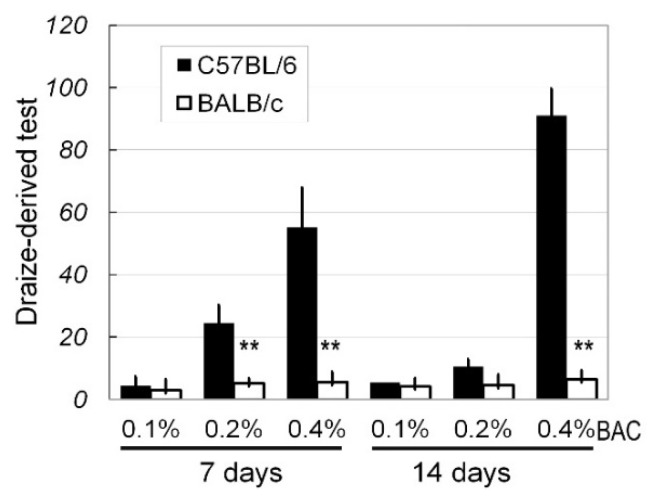
A comparison of ocular surface disruptions by the scores of the Draize-derived test in C57BL/6 and BALB/c mice. Benzalkonium chloride (BAC) eye drops at various concentrations were topically applied twice daily, and the effects were evaluated on Day 7 and Day 14. The criteria for the scores of the Draize-derived test are described in the main text (Materials and Methods). Based on 10 eyes on Day 7 and 10 eyes on Day 14, the results are presented as the mean ± SD. ** *p* < 0.01; **: C57BL/6 vs. BALB/c.

Generally, the effects of different concentrations of BAC (0%, 0.1%, 0.2%, and 0.4%) on the ocular surfaces of C57BL/6 and BALB/c mice presented as stimulus signs and inflammation responses, which were evaluated in detail based on the scores of the Draize-derived test (Figure 1). On Days 7 and 14, 0.1% BAC had no significant effect on the ocular surfaces of both the C57BL/6 and BALB/c mice according to Draize-derived test (Day 7, *p* = 0.28; Day 14, *p* = 0.18). However, treatment with 0.2% and 0.4% BAC resulted in significant differences in the extent of ocular damage between the C57BL/6 and BALB/c mice on Day 7 (for 0.2%, *p* < 0.01; for 0.4%, *p* < 0.01) and 14 (for 0.2%, *p* < 0.01; for 0.4%, *p* < 0.01).

### 2.2. Corneal Edema, Opacity and Neovascularization

**Figure 2:**
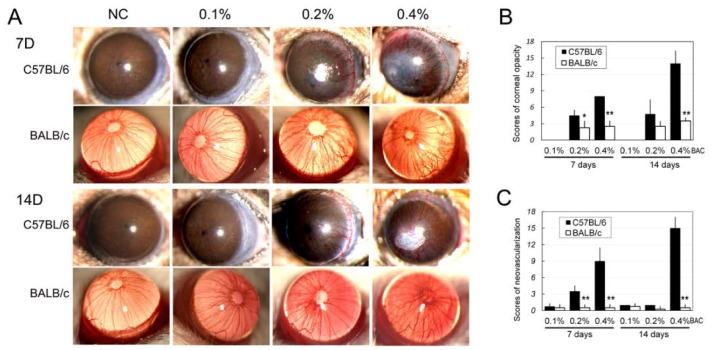
In vivo observations of corneal edema, opacity and neovascularization in mice that received topically applied BAC eye drops. (**A**) Photographs of corneas from C57BL/6 and BALB/mice that were treated with various levels of BAC eye drops for 7 and 14 days. The changes in the corneas were evaluated by scoring: the corneal opacity (**B**); and neovascularization (**C**) following the methods described in the literature (Materials and Methods, *n* = 10). *: *p* < 0.05; **: *p* < 0.01; *, **: C57BL/6 vs. BALB/c. NC: The group received a topical administration of normal saline (NS) as the control.

The results showed that both strains of mice suffered ocular damage, with more obvious damage in the C57BL/6 mice at the same concentration of BAC and the same time point (Figure 2). After administration of BAC, the corneas of the mice gradually developed edema and opacities to various extents. For both strains of mice, the extent of corneal opacity was not significantly different between the 0.1% BAC and control groups on Days 7 and 14. However, with higher amounts of BAC, the corneas of the C57BL/6 mice became more opaque compared to the BALB/c mice. The changes were evaluated using corneal opacity scores, which revealed significant differences between two strains at Day 7 for 0.4% BAC (*p* < 0.01) and 0.2% (*p* < 0.05) and Day 14 for 0.4% BAC (*p* < 0.01) (Figure 2B).

The effects on corneal neovascularization were dose and time dependent (Table 1, Figure 2). No obviously new corneal vessels were observed under the slit-lamp microscope in both the C57BL/6 and BALB/c mice from the control group and the 0.1% BAC group over the 14 days. With the increasing concentration of BAC and drop duration, corneal neovasculars appeared in both the 0.2% and 0.4% BAC-treated groups of both strains on Days 7 and 14. In the mice treated with 0.2% BAC, there was an obvious increase in the number of corneas that developed vessels in the C57BL/6 mice compared to the BALB/c mice (*p* < 0.01). Furthermore, using the scoring method for corneal neovascularization, we found that there were significant differences in the severity of corneal neovascularization between the two strains of mice, with more serious neovascularization in the C57BL/6 mice (Figure 2C).

### 2.3. Disruption of the Corneal Epithelium Induced by Benzalkonium Chloride (BAC) Eye Drops

**Figure 3:**
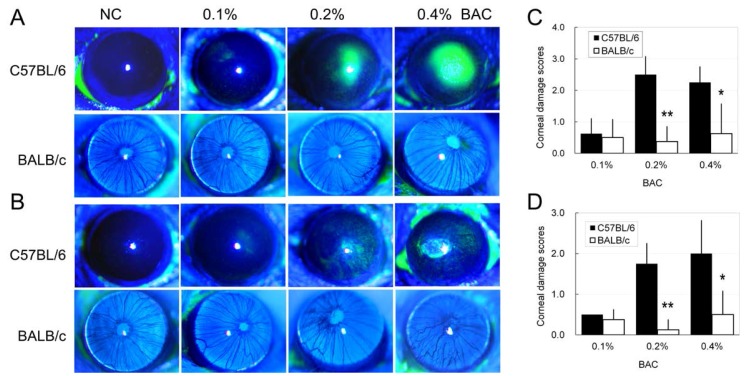
Epithelial disruption in the corneas of C57BL/6 and BALB/c mice induced by topical application of BAC. (**A**,**B**) Photographs of corneas stained with fluorescein sodium. (**C**,**D**) Corneal damage was scored using a subjective method (see Materials and Methods). Evaluations were conducted on: Day 7 (**A**,**C**); and Day 14 (**B**,**D**). *n* = 10. *: *p* < 0.05; **: *p* < 0.01; *, **: C57BL/6 vs. BALB/c.

Disruptions of corneal epithelium were observed in some animals when examined by slit-lamp microscopy and stained with fluorescein sodium (FLS) (Figure 2 and Figure 3). In the control groups of both the C57BL/6 and BALB/c strains, no FLS staining in the corneal epithelium was observed on Days 7 and 14, and the scores of FLS staining in these mice were considered to be 0. Epithelial damage, i.e., FLS staining and corneal ulcers, developed after BAC treatment to different extents. More severe epithelial damage was observed in the C57BL/6 mice compared with the BALB/c mice that were treated with the same BAC concentration and at the same time point. Additionally, the damage was more obvious on Day 7 than Day 14 for the animals under the same treatment. There were significant differences in the FLS staining scores between the C57BL/6 and BALB/c mice on Day 7 (for 0.2%, *p* < 0.01; for 0.4% BAC, *p* < 0.05) and Day 14 (for 0.2%, *p* < 0.01; for 0.4% BAC, *p* < 0.05).

### 2.4. Histological Changes in the Cornea after BAC Treatment

**Figure 4:**
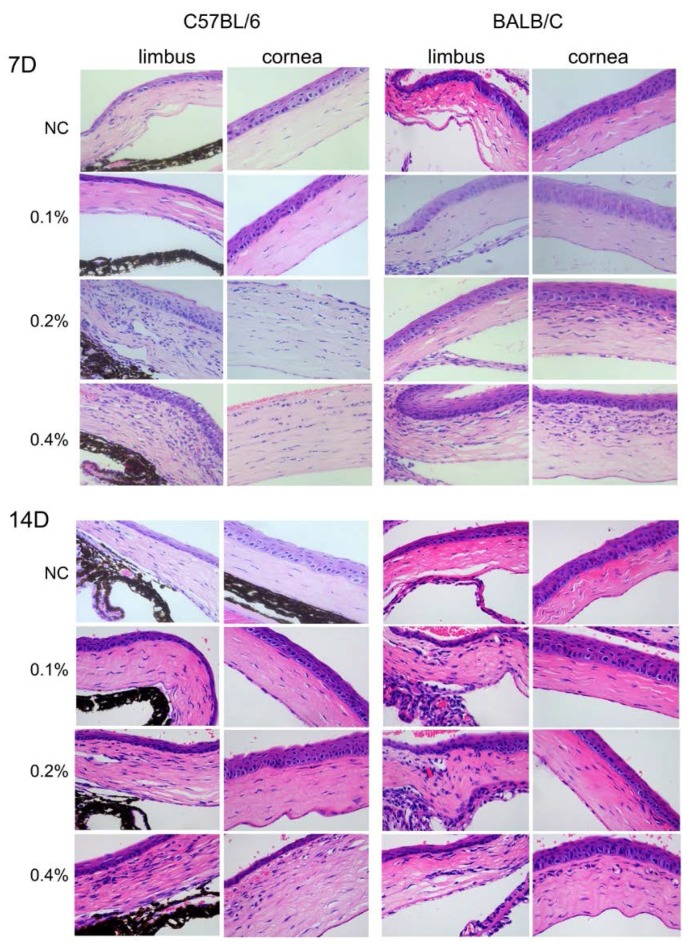
Histological evaluation of corneal alterations induced by the administration of BAC eye drops for 7 and 14 days. Hematoxylin/eosin staining of corneal sections shows disruption of the epithelial layer of the cornea, neovascularization, and infiltration of inflammatory cells in the peripheral and central cornea. Strong responses were observed in tissue sections from the C57BL/6 mice (vs. the BALB/c mice), in sections from mice that received a high dose of BAC (in the following order: 0.4% > 0.2% > 0.1% > NC), and in sections obtained on Day 7 (vs. Day 14). All pictures are at the same magnification (400×).

The results of the histologic examinations are shown in Figure 4. The cornea and limbus of the control group of C57BL/6 and BALB/c mice presented with a visible and relatively thick epithelium containing small cubic basal cells and large squamous cells undergoing desquamation. The stromal fibers were orderly and normally arranged with few keratocytes. No obvious signs of inflammation or new vessels in the limbus and cornea were observed in both the C57BL/6 and BALB/c mice that were treated with 0.1% BAC on Days 7 and 14. Nevertheless, treatment with both 0.2% and 0.4% BAC resulted in stromal neovascularization and infiltration of inflammatory cells on Days 7 and 14 (Figure 4) in C57BL/6 and BALB/c mice. Treatment with 0.2% and 0.4% BAC resulted in substantially more neovascularization and infiltration of inflammatory cells in the limbus and central cornea of the C57BL/6 mice than the BALB/c mice at the same point time. In the C57BL/6 mice treated with 0.2% and 0.4% BAC, the superficial epithelial layers of the cornea were disrupted and desquamated, while no change or fewer changes were observed in the control and 0.1% BAC groups, respectively. The photographs of the corneal blood vessels (Figure 2) and corneal FLS staining (Figure 3) are in accordance with the results of the histological analyses.

### 2.5. Goblet Cell Changes in Conjunctiva Treated with BAC

**Figure 5:**
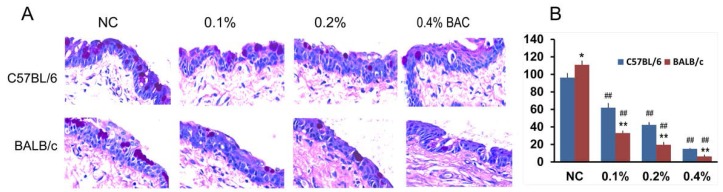
Evaluation of goblet cell number in the conjunctiva of mice that were treated with BAC eye drops: (**A**) Periodic acid-Schiff staining of the conjunctiva showing goblet cells within the epithelial layer of the C57BL/6 and BALB/c mice. Photos were magnified at 400×. (**B**) Quantification of goblet cells in the conjunctiva of the various treatment groups. Three different views of each section for one eye were randomly selected for counting, and the average of five eyes was calculated (cells/high-power visual field, 400×). *: *p* < 0.05; **, ##: *p* < 0.01; *, **: C57BL/6 vs. BALB/c; ##: test point vs. NC.

Different numbers of goblet cells were observed in the normal conjunctiva of both the C57BL/6 and BALB/c mice (*p* < 0.05). After treatment with BAC eye drops over 14 days, the number of conjunctival goblet cells decreased in a dose-dependent manner (Figure 5), which was consistent with the corneal FLS staining (Figure 3B) in both strains of mice. Moreover, the numbers of goblet cells in the conjunctiva of the BABL/c mice that were treated with 0.1%, 0.2% and 0.4% BAC were significantly lower than that of the C57BL/6 mice (*p* < 0.01, for all pairs). 

### 2.6. Changes of Inflammatory Factors in the Corneas after BAC Irritation

**Figure 6:**
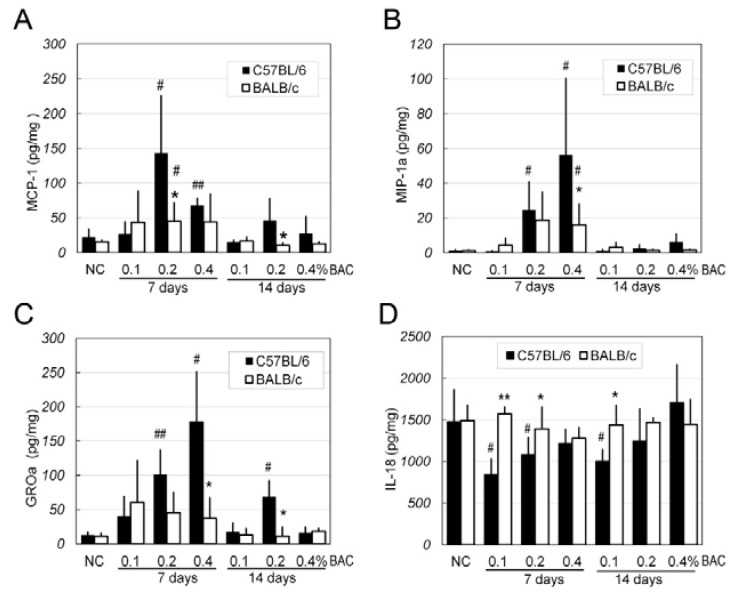
Quantification of inflammatory factors in corneal extracts by multiplex immunoassay. (**A**) monocyte chemoattractant protein 1 (MCP-1); (**B**) growth-regulated protein alpha (MIP-1a); (**C**) macrophage inflammatory protein-1 alpha (GROa); and (**D**) IL-18 levels are shown in the bar graphs representing the different treatments applied to the corneas of the two strains of mice. Data are expressed as the mean ± SD; *n* = 5, *, #: *p* < 0.05; **, ##: *p* < 0.01; *, **: BALB/c vs. C57BL/6; #, ##: test point vs. NC.

Using multiplex immunoassay technology, four inflammatory factors were measured in the corneas of mice that were treated with BAC eye drops over 7 and 14 days (Figure 6). Three of the chemokines, i.e. monocyte chemoattractant protein 1 (MCP-1), growth-regulated protein alpha (GROa) and macrophage inflammatory protein-1 alpha (MIP-1a), showed similar expression patterns, e.g., increasing in a dose-dependent manner and decreasing by Day 14, and a stronger response to BAC was observed in the C57BL/6 strain than the BALB/c strain in all experiments. However, the IL-18 levels were high in the corneas of the both strains (around 1500 pg/mg protein) and did not change in any of the BALB/c groups. In the C57BL/6 strain, the levels were decreased in the corneas of the group that was administered eye drops containing 0.1% BAC.

## 3. Discussion

BAC has been used as a preservative in ophthalmic agents for a long time and its toxicity to ocular surfaces has resulted in concern continually. Several studies have evaluated the toxicity of BAC in in vitro cell models and in vivo animal models, as well as in patients [10,19,28,29]. In vivo animal studies have revealed that the application of BAC results in damages such as tear film instability, cornea and conjunctival epithelial disruption, corneal neovascularization and inflammatory responses in mice, rats, and rabbits [10,14,30]. Of the various animal models, two strains of mice, i.e., C57BL/6 and BALB/c, have been widely used and treated in similar manners to create models of dry eye that exhibit the instability of the tear film and inflammation of the ocular surfaces [10,27]. Furthermore, Galletti and colleagues compared the effects of BAK on conjunctival tolerance and found no differences in the induction of tolerance/immunity between the BALB/c and the C57BL/6 mice [31]. Until now, however, no studies have reported a difference in the ocular responses to BAC irritation between these two strains of mice. In this study, we evaluated the toxic characteristics of the ocular surfaces of the two strains of mice and found that BAC induced more serious injuries to the ocular surface with peaks during the first week in the C57BL/6 mice than in the BALB/c mice. Our results suggest that the strong responses in the corneas and conjunctivas of these two strains of mice make them suitable for mimicking serious ocular damage, especially the C57BL/6 strain.

The concentration of BAC required to damage the ocular surfaces varies in different studies, with greater differences observed between the in vivo and in vitro studies. Generally, the BAC concentration of the eye drops ranged from 0.004% to 0.01%, which may lead to damage of ocular surfaces in patients who use them long term [1]. Furthermore, the prevalence of discomfort or corneal/conjunctival disruptions in patients was dose dependent, increasing with the amount of preserved eye drops administered [32]. For in vitro cell studies, the BAC levels in culture media were much less than those in the in vivo animal experiments and as low as 0.0003% in some studies [33,34]. In studies involving animal models in which dry eye and toxicity tests were frequently conducted, the concentration of BAC that was topically applied varied case by case from 0.01% to 0.5%, with lower concentrations corresponding to longer durations of observation [35,36]. However, 0.2% BAC was frequently administered to dry eye models. In the current study, we administered BAC eye drops at four concentrations (0%, 0.1%, 0.2%, and 0.4%), which covered the ranges that were frequently used in other studies. Dose-dependent effects were observed in both animals, and they were stronger in the C57BL/6 mice during the first seven days. 

In addition to the observations of in vivo disruptions and pathological alterations, we also examined the expression of several inflammatory factors using a multiplex immunoassay. We found that three chemotactic factors, i.e., MCP-1 (CCL2), GROa (CXCL1), and MIP-1a (CCL3), increased after seven days of treatment with BAC. These factors have potent chemotactic activity in monocytes, neutrophils, and eosinophils, either together or separately (available online at: http://www.uniprot.org). These results are consistent with the in vivo and pathological findings, including stronger inflammatory responses in the C57BL/6 mice (vs. BALB/c mice) and during the first week (vs. second week). Denoyer et al. have documented that the expression of CX3CL1, a member of the CX3C-chemokine subfamily that binds to its specific receptor CX3CR1 and induces the migration and activation of CX3CR1-bearing cells such as T cells, natural killers (NKs), monocytes, monocyte-derived macrophages and dendritic cells, was increased in the conjunctiva of patients and mice treated with BAC-containing eye drops [9]. Our current results, as well as recent reports by Kim, provide evidence of the high expression of chemokines in corneas treated with BAC [24]. In the present study, we also found that IL-18, an inducer of interferon gamma, was highly expressed in both strains of mice. After administration of BAC, no differences in corneal IL-18 levels were detected between the normal BALB/c mice and the BAC-treated BALB/c mice. The present results suggested that chemokines might contribute to the BAC-induced inflammation response of the cornea, and their mechanism of action will be further investigated in our future study.

Our results are in agreement with other studies that report heavy inflammation appearing during the first week and later decreasing, even when BAC irrigation was continuously applied, because similar intensive treatments, high BAC concentrations and/or quick dripping were used [19,37]. All observations, including those involving in vivo changes, pathological tissues, and inflammatory factors, showed consistent peak alterations. Similarly, rabbit corneal changes tended to recover within two weeks of the successful establishment of a dry eye model [4]. However, the studies that used lower concentrations of BAC reported increasing damage to the cornea and conjunctiva over two to four weeks, leading to the outcome of dry eye [27]. The effects on the peak injuries on the seventh day have yet to be determined.

## 4. Materials and Methods

### 4.1. Animals

Female mice, including 80 BALB/c mice and 80 C57BL/6 mice, aged 6–8 weeks and weighing 15–20 g (purchased from the Guangdong Provincial Center for Animal Research, Guangzhou, China) were used for this study. In the Ophthalmic Animal Laboratory of the Zhongshan Ophthalmic Center, these mice were quarantined and acclimatized for a week before the experiments were initiated. The mice were free of clinically observable ocular surface disease and kept in a standardized environment throughout the study as follows: a constant temperature of 23 ± 1 °C, a relative humidity of 60% ± 5%, and alternating 12 h light–dark cycles (8:00 a.m. to 8:00 p.m.). All experiments and animal care procedures were conducted in accordance with the Association for Research in Vision and Ophthalmology (ARVO) Statement for the Use of Animals in Ophthalmic and Vision Research under the supervision of a health authority-accredited staff member for animal care and management. In addition, the research protocol was approved by the Animal Care Committee of the Zhongshan Ophthalmic Center at Sun Yat-sen University (approval ID: 2013-077, Guangzhou, China, 8 October 2013).

### 4.2. BAC Treatment

Both C57BL/6 and BALB/c mice were randomly assigned to four groups with 20 mice each, and the right eye of each mouse was chosen for the experiment. Each group received a topical administration of different concentrations of BAC (Sigma-Aldrich, St. Louis, MO, USA) solution: 0.1%, 0.2%, or 0.4% in normal saline (NS) or NS as the control. The mice were treated twice daily (8:00 a.m. and 8:00 p.m.) for 14 consecutive days. The mice were gently restrained, and 10 μL BAC solution or NS was administered by micropipette into the inferior conjunctival sac of the eye. To ensure adequate contact time for the solution on the ocular surface and prevent aggressive blinking during administration of the solution, which may cause variability in the ocular surface contact of the solution, the eyes were held open for 60 s.

The ocular surface changes (including persistent corneal new vessels, corneal edema and opacity) and fluorescein staining (corneal epithelial damage) were evaluated using a slit-lamp microscope (Sun Kingdom Medical Instruments, Chongqing, China) on Days 0, 7 and 14. On the 7th and 14th days, 10 mice per group were sacrificed, and the ocular global specimens were carefully dissected and harvested for histological analysis as well as periodic acid-Schiff (PAS) (*n* = 5) and cytokine detection (*n* = 5) following the methods described below.

### 4.3. Ocular Surface Alterations and the Draize Test

On Days 0, 7 and 14, the animals were anesthetized by intraperitoneal injection of 10% chloral hydrate (300 mg/kg) and observed under the slit-lamp microscope without topical anesthesia. The ocular irritation was scored according to the Draize test [5,38], and the maximum total score possible was 110 (cornea, 80; iris, 10; conjunctiva, 20).

### 4.4. Measurement of Corneal Neovascularization and Opacity

At every time point, corneal neovascularization was examined under general anesthesia and scored by a single masked ophthalmologist with the slit lamp following the methods reported by Pauly and colleagues [5]. Briefly, the cornea was divided into four quadrants, which were scored separately. The neovascularization area was scored as follows: 0, no vessels growing into the clear cornea; 1, one quarter (or less) but not zero; 2, between one quarter and one half; 3, between one half and three quarters; and 4, between three quarters and the entire surface of the cornea. The final scores of corneal neovascularization were calculated by summing the scores of the four quadrants (total, 16 points).

The severity of corneal opacity was analyzed using the slit lamp according to a previously described method [5] and was scored as follows: 1, scattered or diffuse area with details of the iris visible; 2, easily discernible translucent areas with details of the iris slightly obscured; 3, opalescent areas with no details of the iris visible, size of pupil barely discernible; and 4, opaque, iris not visible. The area of opacity was scored as follows: 1, one quarter (or less) but not zero; 2, greater than one quarter but less than one half; 3, greater than one half but less than three quarters; and 4, greater than three quarters, up to the entire area. The maximum total score possible was 16.

### 4.5. Evaluation of Fluorescein Staining

Two microliters of 0.5% fluorescein sodium (FLS) solution were instilled into the conjunctival sac of each mouse. Ninety seconds later, the eyes were rinsed with NS. Corneal epithelial damage was examined under a slit-lamp microscope with a cobalt blue filter. The extent of corneal damage (width and intensity of the area with fluorescein uptake) was scored according to the following scale [5]: 0, no staining; 0.5, slight punctate staining; 1, diffuse punctate staining; 2, diffuse staining covering less than one third of the cornea; 3, diffuse staining covering more than one third of the cornea; and 4, staining covering more than two thirds of the cornea.

### 4.6. Histologic Analysis and Assessment of Conjunctival Goblet Cells 

On Days 7 and 14 following the BAC solution treatment, 10 mice in each group were sacrificed, and the eyeballs were gently dissected. Five ocular specimens in each group were fixed in 10% formalin and then embedded in paraffin. The tissues sections were stained with hematoxylin and eosin (H&E) for histomorphologic analysis, and with periodic acid-Schiff (PAS) for conjunctival goblet cells [24,39]. After PAS staining with a commercially available kit (395B-1KT, Sigma-Aldrich) following the manufacturer’s instructions [24], the number of goblet cells in the conjunctivas on Day 14 was counted under a microscope (Olympus, Tokyo, Japan). Three different portions of each specimen were randomly selected for counting, and the average was calculated (cells/high-power visual field, 400×).

### 4.7. Detection of Corneal Inflammatory Factors 

The dissected corneal tissues from each eye (*n* = 5) were homogenized and centrifuged (12,000× rpm, 4 °C for 15 min) to extract proteins using the cold CelLytic^™^ MT Cell Lysis Reagent (Sigma-Aldrich, St. Louis, MO, USA) following the manufacturer’s instructions. The concentrations of cytokines in the supernatants (50 μL) were analyzed using the ProcartaPlex^®^ Multiplex Immunoassay (Affymetrix, eBioscience, Santa Clara, CA, USA), which uses Luminex technology (multianalyte profiling beads) to simultaneously detect and quantify multiple protein targets in a sample. All of the experiments were conducted in accordance with the manufacturers’ instructions. Total protein concentrations were determined by the bicinchoninic acid (BCA) method using BSA (Shanghai Shengzheng Biot. Co., Ltd, Shanghai, China) as a protein standard [40]. 

### 4.8. Statistical Analysis

Statistical analysis was performed with SPSS software (version 18.0, SPSS Inc., Chicago, IL, USA). Independent sample *t*-tests and one-way ANOVA were applied for comparisons between groups. For all statistical tests, *p*-values less than 0.05 were considered statistically significant.

## 5. Conclusions

Our results demonstrate that topical BAC application can significantly disrupt the ocular surfaces of C57BL/6 and BALB/c mice, and the effects are more severe in the former. The BAC-induced ocular surface damage, e.g., corneal epithelial disruption, stromal neovascularization and infiltration of inflammatory cells, occurred in a dose-dependent manner and was more serious on Day 7 than on Day 14. The results of the histological analyses and the assessment of chemokine levels were in accordance with the in vivo changes to the ocular surfaces. The administration of BAC eye drops has recently been used for shorter durations in animal models such as the dry eye model. Because serious complications were observed in the corneal and conjunctival tissues, the topical application of high doses of BAC over short durations may be not suitable for mimicking the changes associated with dry eye in mice, especially in the C57BL/6 strain.

## Figures and Tables

**Table 1:** Prevalence of corneal neovascularization in the two strains of mice.

BAC%	C57BL/6 (%) #		BALB/c (%) #	
	7 Days	14 Days	7 Days	14 Days
0.10	0 (0.0)	0 (0.0)	0 (0.0)	0 (0.0)
0.20 *	9 (90.0)	10 (100.0)	4 (40.0)	6 (60.0)
0.40	10 (100.0)	10 (100.0)	10 (100.0)	10 (100.0)

# 10 eyes from 10 animals were examined for each observed point. * Significant differences were observed between the C57BL/6 and BALB/c mice at Days 7 and 14, respectively.
